# Le carcinome épidermoïde palpébral: prise en charge d'une forme évoluée chez un sujet jeune

**DOI:** 10.11604/pamj.2014.19.275.5610

**Published:** 2014-11-12

**Authors:** Abdelmoughit Echchaoui, Malika Benyachou

**Affiliations:** 1 Service de Chirurgie Réparatrice et Plastique, CHU Ibn Sina, Université Med V, Souissi, Rabat, Maroc

**Keywords:** Carcinome épidermoïde, paupière, exentération, reconstruction, squamous cell carcinoma, eyelid, exenteration, reconstruction

## Image en medicine

Les carcinomes palpébraux représentent une pathologie relativement fréquente. Contrairement au carcinome basocellulaire, le carcinome épidermoïde est caractérisé par une agressivité locorégionale rapidement extensive et doué de potentiel métastatique. La prise en charge commence par la prévention des facteurs de risques en particulier l'exposition solaire, le diagnostic précoce et par une attitude thérapeutique rigoureuse, qui permettent d’éviter une évolution catastrophique locale ou à distance. Nous rapportons l'observation d'un patient de 42 ans, agriculteur, tabagique chronique présentant depuis un an une cécité unilatérale gauche, l'examen clinique montrait une déformation orbitaire avec une infiltration palpébrale par une lésion ulcéro-bourgeonnante saignante au contact, à point de départ canthal externe, l'examen des aires ganglionnaires était libre, la biopsie était en faveur d'un carcinome épidermoïde invasif. Le scanner crânio-facial objectivait un envahissement du globe oculaire et de la graisse extra et intra-conique. Le patient avait bénéficié d'une exentération totale, les limites d'exérèse à l'examen extemporané ainsi que pour l'examen histologique postopératoire étaient saines, la reconstruction était faite par le lambeau du muscle temporal avec greffe de peau totale, ce qui a permis une bonne couverture cutanée et une radiothérapie externe précoce. Une épithèse oculo-palpébrale a été proposée ultérieurement.

**Figure 1:**
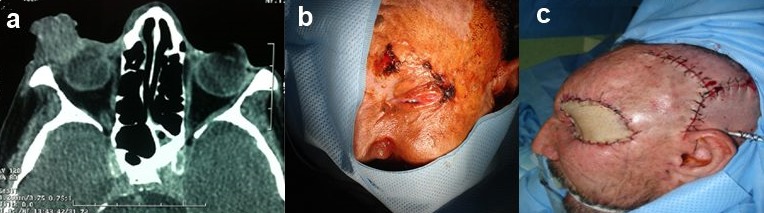
(A) scanner crânio-facial en coupe axiale montrant un carcinome du canthus externe avec envahissement orbitaire; (B) aspect préopératoire du carcinome épidermoïde du canthus externe avec envahissement orbitaire; (C) aspect postopératoire immédiat après reconstruction de la cavité d'exentération par transposition du muscle temporal et greffe de peau totale

